# Ciprofloxacin-resistant *Salmonella* Kentucky in Travelers

**DOI:** 10.3201/eid1210.060589

**Published:** 2006-10

**Authors:** François-Xavier Weill, Sophie Bertrand, Françoise Guesnier, Sylvie Baucheron, Patrick A.D. Grimont, Axel Cloeckaert

**Affiliations:** * Institut Pasteur, Paris, France;; † Scientific Institute of Public Health, Brussels, Belgium;; ‡ Institut National de la Recherche Agronomique, Nouzilly, France

**Keywords:** Salmonella, ciprofloxacin resistance, human, travelers, letter

**To the Editor:** Ciprofloxacin is the treatment of choice of severe nontyphoidal *Salmonella* infections in adults. Resistance to ciprofloxacin has been found exceptionally in nontyphoidal *Salmonella enterica* isolates and only in serotypes Typhimurium, Choleraesuis, and Schwarzengrund (*1**–**8*). Such isolates have been collected from humans and animals in Europe, Asia, and North America.

We report the emergence of ciprofloxacin-resistant isolates of *S*. Kentucky since 2002 in French travelers returning from northeast and eastern Africa. From 2000 through 2005, 197 *S*. Kentucky isolates from humans (1 per patient) were serotyped, from 69,759 total *S. enterica* isolates serotyped at the French National Reference Centre for *Salmonella*. Antimicrobial drug susceptibility was determined for 186 isolates by the disk-diffusion method with 32 antimicrobial drugs, as previously described (*9*). Resistance to several drugs, amoxicillin (18%), gentamicin (16%), nalidixic acid (21%), sulfonamides (24%), and tetracycline (24%), has been observed from 2000 through 2005.

A total of 17 (9%) ciprofloxacin-resistant *S*. Kentucky strains were isolated. A resistant isolate that was untypable by conventional serotyping (rough) but that had a pulsed-field gel electrophoresis (PFGE) profile associated with serotype Kentucky, was included in this study. Ciprofloxacin MIC levels in these isolates, determined by standard agar doubling dilution as previously described (*2*), were 4–16 mg/L. The first ciprofloxacin-resistant strain was isolated in December 2002 from a French tourist who had gastroenteritis during a Nile cruise in Egypt. In 2004 and 2005, 17 ciprofloxacin-resistant isolates were identified in unrelated adults who lived in different cities of France at different times of the year. The 16 patients we contacted acquired the infection during or immediately after travel to Egypt (10 patients), Kenya and Tanzania (3), or Sudan (1). In 2 cases, gastroenteritis occurred 2 months after travel to Egypt. None of the investigated cases were fatal or life-threatening.

The 18 ciprofloxacin-resistant isolates (17 serotype Kentucky and 1 rough) displayed various susceptibility patterns, from single resistance to quinolones to multiple resistance (up to 9 antimicrobial agents). To identify mutations responsible for ciprofloxacin resistance, the quinolone resistance–determining regions (QRDRs) of *gyrA*, *gyrB*, *parC*, and *parE* were amplified by PCR and sequenced as described previously (*3**,**9*), except that different forward primers for *gyrB* (5´-TTATCGACGCCGCGCGTGCGC-3´) and *parE* (5´-CGCGTAACTGCATCGGGTTC-3´) were used. The 18 ciprofloxacin-resistant isolates had different double mutations in *gyrA* leading to amino acid substitutions, Ser83Phe and Asp87Gly (8 isolates), Ser83Phe and Asp87Asn (*5*), and Ser83Phe and Asp87Tyr (*5*), but had identical mutations in *parC* (resulting in Ser80Ile). An additional substitution was observed in ParC, Thr57Ser. This substitution, however, did not appear to be associated with quinolone resistance because it was also identified in nalidixic acid–susceptible isolates. No isolates had substitutions in the QRDRs of GyrB and ParE. All isolates tested by PCR for the plasmid-mediated quinolone resistance–conferring gene *qnrA* (*9*) were negative. In the presence of the efflux pump inhibitor Phe-Arg-β-naphthylamide, the MICs of ciprofloxacin were reduced from 4-fold to 16-fold, which suggests that an active efflux mechanism was present (*2*). The involvement of the AcrAB-TolC efflux system was determined by measuring AcrA expression with a method previously described (*5*). A moderate production of AcrA (3- to 4-fold increase when compared with the baseline production of AcrA in reference strain 98K) was observed in all but 1 ciprofloxacin-resistant isolate. This isolate overproduced (6-fold) AcrA, which correlated with a higher ciprofloxacin MIC (16 mg/L).

The 18 ciprofloxacin-resistant isolates and 14 ciprofloxacin-susceptible *S*. Kentucky isolates used for comparison were genotyped by PFGE with *Xba*I restriction and PulseNet's running conditions, as described previously (*9*). Each profile that differed by >1 clear band >50 kb was considered a distinct profile. The 18 resistant isolates displayed 9 profiles that differed by 1 to 3 bands (Dice correlation coefficient 55%) (Figure). Profile X1c was predominant (7 [39%] of 18). The 6 pansusceptible isolates tested displayed 5 different patterns unrelated to those of resistant isolates. Use of a second restriction enzyme, *Spe*I, for the resistant isolates of X1 cluster enhanced discrimination. No clear correlations between combined PFGE patterns, *gyrA* mutations, and probable country of infection were observed.

**Figure:**
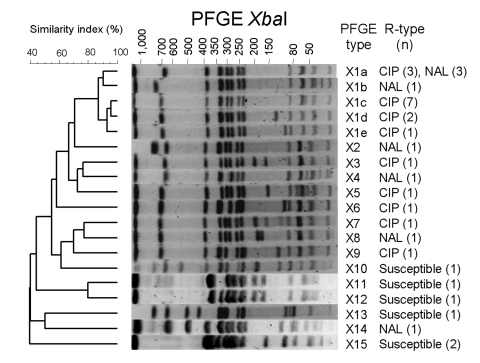
Dendrogram generated by BioNumerics version 4.1 (Applied Maths, Sint-Martens-Latem, Belgium) showing the results of cluster analysis on the basis of XbaI pulsed-field gel electrophoresis (PFGE) of Salmonella enterica serotype Kentucky isolates. Similarity analysis was performed by using the Dice coefficient, and clustering was performed by the unweighted pair-group method with arithmetic means with an optimization parameter of 0.5% and a 0.5% band position tolerance. The different PFGE profiles, the phenotypes of resistance to quinolones (R-type), and corresponding number of isolates are indicated. CIP, ciprofloxacin; NAL, nalidixic acid.

Since *S*. Kentucky is infrequently isolated from human, animal, or environmental sources in France, these isolates must have been acquired abroad. Unfortunately, no investigations have been thus far conducted to identify the source of the contamination in probable countries of infection. Poultry products may be of particular interest because poultry is the main animal reservoir of *S*. Kentucky. Another possible source in East Africa is pork because a recent report identified quinolone-resistant (ciprofloxacin MIC >0.125 mg/L) *S*. Kentucky isolates in slaughtered pigs in Ethiopia (*10*). After identifying the source of the contamination, appropriate control measures should be implemented in the affected countries to control the spread of these isolates.
